# Representations of specific acoustic patterns in the auditory cortex and hippocampus

**DOI:** 10.1098/rspb.2014.1000

**Published:** 2014-09-22

**Authors:** Sukhbinder Kumar, Heidi M. Bonnici, Sundeep Teki, Trevor R. Agus, Daniel Pressnitzer, Eleanor A. Maguire, Timothy D. Griffiths

**Affiliations:** 1 Institute of Neuroscience, Medical School, Newcastle University, Newcastle upon Tyne NE2 4HH, UK; 2 Wellcome Trust Centre for Neuroimaging, Institute of Neurology, University College London, 12 Queen Square, London WC1N 3BG, UK; 3 Laboratoire des Systèmes Perceptifs, CNRS UMR 8248, and Ecole Normale Superieure, Paris, France

**Keywords:** acoustic patterns, fMRI, auditory cortex, multi-voxel pattern analysis, hippocampus

## Abstract

Previous behavioural studies have shown that repeated presentation of a randomly chosen acoustic pattern leads to the unsupervised learning of some of its specific acoustic features. The objective of our study was to determine the neural substrate for the representation of freshly learnt acoustic patterns. Subjects first performed a behavioural task that resulted in the incidental learning of three different noise-like acoustic patterns. During subsequent high-resolution functional magnetic resonance imaging scanning, subjects were then exposed again to these three learnt patterns and to others that had not been learned. Multi-voxel pattern analysis was used to test if the learnt acoustic patterns could be ‘decoded’ from the patterns of activity in the auditory cortex and medial temporal lobe. We found that activity in planum temporale and the hippocampus reliably distinguished between the learnt acoustic patterns. Our results demonstrate that these structures are involved in the neural representation of specific acoustic patterns after they have been learnt.

## 1. Introduction

As humans we are constantly bombarded with sounds, many of which can be identified and assigned a semantic label. However, before a label is assigned, the auditory system must first learn a ‘template’ corresponding to the specific acoustic structure. Although a number of brain imaging studies [1–4] have highlighted the brain system which represents the meaning of sounds, key questions remain as to how the brain learns novel acoustic patterns, and whether a specific mechanism exists for the storage of acoustic patterns *per se*.

Behavioural studies have shown that repeated presentation of complex acoustic patterns results in the learning of templates. In a series of studies [5–7], subjects were presented with either a 1 s sample of white noise (noise condition, N) or two identical and seamlessly abutting 0.5 s samples of white noise (repeated noise condition, RN). For both RN and N stimuli, samples of white noise were generated anew from trial to trial. However, without this being mentioned to subjects, there was in fact a third type of trials: one particular exemplar of repeated noise was presented over several trials, randomly interspersed throughout an experimental block. These trials (reference repeated noise, RefRN) were thus initially drawn from the same process as RN but, unlike RN, they were acoustically identical across several trials. The task assigned to subjects was to report the presence or the absence of repetition within the noise: after each trial, they pressed one button (‘yes’) if they heard a repetition and another button (‘no’) if not. The main result was that performance was considerably better for the RefRN stimuli than for the RN stimuli. Since the only difference between RefRN and RN was that RefRN was heard over several trials, the improved performance could be attributed to the learning of a template for RefRN. The learning process was fast, robust and unsupervised. Furthermore, learning appeared to be largely implicit: even though this was not systematically quantified, the majority of subjects seemed unaware that the same sound had been presented over different trials. Using a different paradigm, McDermott *et al*. [8] showed that when a fixed acoustic pattern mixed with other randomly chosen acoustic patterns is repeatedly presented, segregation of the fixed acoustic pattern from other patterns could be achieved. This is also consistent with a learning of random patterns through repeated exposure.

Given the suggested importance of repeated exposure for auditory learning, we sought specific brain representations of acoustic patterns following repeated exposure. We used high-resolution functional magnetic resonance imaging (fMRI) and multi-voxel pattern analysis (MVPA). Subjects were first exposed to three acoustic patterns, among many other highly similar patterns, to induce learning of the specific patterns. During subsequent fMRI scanning, subjects were again exposed to the three learned spectro-temporal patterns, along with other novel patterns that shared their average spectro-temporal characteristics. MVPA was used to test if the three exemplars could be ‘decoded’ from the patterns of activity in the brain.

Given evidence for the involvement of primary auditory cortex in storing long-term representations of specific auditory experiences [9–11], we hypothesized that Heschl's gyrus (HG), which contains primary auditory cortex, would be involved. We also predicted that areas of non-primary/associative areas of auditory cortex would also be recruited. Specifically, we predicted engagement of planum temporale (PT) which has been hypothesized to be involved in the generation of acoustic ‘templates’ at a stage before semantic processing [12] and superior temporal sulcus (STS), which has been shown to store long-term memories for sounds [13,14]. We also hypothesized that structures in the medial temporal lobes (MTLs) would be crucial. In addition to hippocampus (HC), which has been shown to have a role in long-term memory for sounds [15,16], we also speculated that entorhinal/perirhinal cortex (EPC) and parahippocampal cortex (PHC), both of which receive dense input from the non-primary auditory areas [17], may be involved.

## 2. Material and methods

### (a) Participants

Seven healthy subjects (two females, mean age = 22.85 years, s.d. = 1.67 years, range = 20–24 years) with no prior history of neurological and psychiatric disorders participated in the study. All subjects completed a consent form and were paid for their participation.

### (b) Stimuli

**Figure 1.**
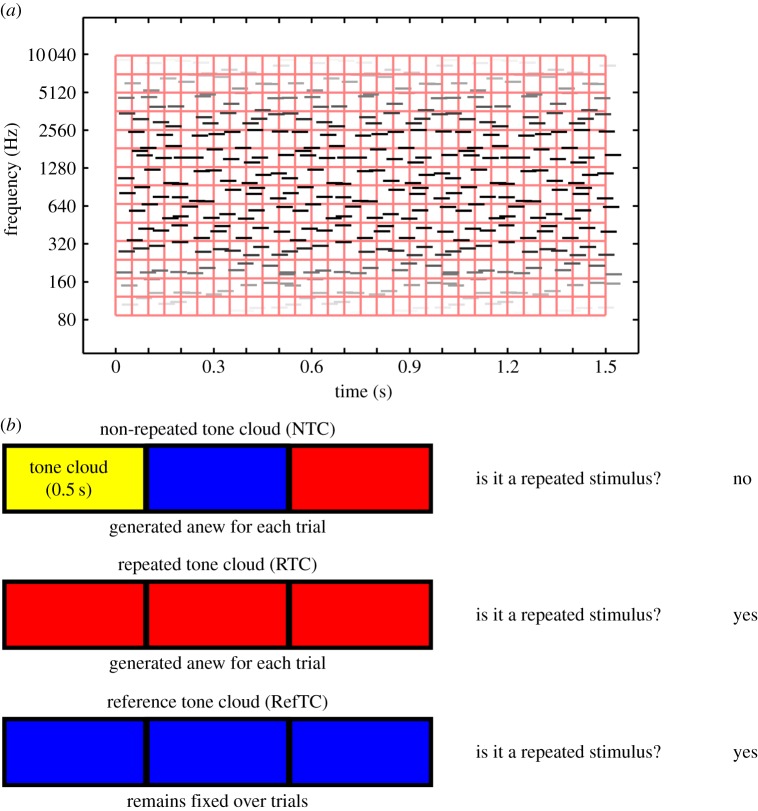
(*a*) Schematic of a tone cloud stimulus. Each stimulus consisted of brief tone pips at random frequencies (with two channels per octave) and random onset times. (*b*) Illustration of the stimuli and task. The NTC stimuli were formed by concatenating three (0.5 s each) segments of tone cloud. The RTC consisted of three repetitions of a single tone cloud segment of 0.5 s. Both NTC and RTC were generated anew for each trial. The RefTC also consisted of three repetitions of a single segment of 0.5 s but, importantly, the same stimulus was used for all trials. In the experiment, subjects were presented with a single stimulus and the task was to detect repetitions in the stimulus by pressing one button if repetitions were detected and another if no repetitions were detected.

The stimuli were ‘tone clouds’. These are noise-like stimuli with a coarser spectro-temporal structure than white noise that allows them to be perceived easily under scanning conditions [18]. The stimuli consisted of multiple brief tones (50 ms) at random frequencies spanning a range from 100 to 10 000 Hz, with random onset times. Specifically, the time-frequency plane was divided into non-overlapping frequency channels and time windows (figure 1). For each cell in the resulting grid, a pure tone was generated with random onset time and random frequency within the cell. This allows the matching of long-term spectrum and temporal envelope for all tone clouds on average. There were two frequency channels per octave and 50 ms per time window. The stimuli were 1.5 s in duration and comprised either three identical and contiguous 0.5 s tone clouds (repeated tone cloud, RTC) or three different and contiguous 0.5 s tone clouds (non-repeated tone cloud, NTC). Both RTC and NTC stimuli were generated anew for each trial. Without the subjects’ knowledge, however, one exemplar of a repeated tone cloud (reference tone cloud, RefTC) remained the same from trial to trial. That is, for the RefTC stimulus not only the same burst of tone cloud was repeated (three times) within a trial, but also the exact same exemplar reoccurred over several trials. The trials consisting of RefTC stimuli were presented randomly among trials of RTC and NTC. A RefTC stimulus was drawn from the same statistical process as any other RTC; the only difference was that the same exemplar of RefTC was presented across different trials.

### (c) Training

The training paradigm was similar to that employed in [5]. A single trial consisted of the presentation of a single stimulus (either of RefTC, RTC or NTC category, chosen randomly). After listening to the stimulus, the task of the subject was to detect repetitions in the stimulus by pressing one button if the tone cloud stimulus repeated within a trial and pressing another if no repetition was detected (figure 1). A single training block consisted of 20 trials each of RefTC, RTC and NTC stimuli. Three separate blocks of training were used, each consisting of a different exemplar of RefTCs. The RefTC trials were pseudorandomly mixed with trials of RTC and NTC such that RefTC stimuli never occurred on successive trials. All training occurred inside the MRI scanner while it was running in order to create the same conditions during learning as during subsequent testing. Given the repeated exposure to the identical spectro-temporal structure of the RefTC stimuli (compared to the variable spectro-temporal structure from trial to trial of the RTC), subjects were expected to form memories of the RefTCs which would be reflected in a better performance on repetition detection for RefTC compared to RTC.

### (d) Testing during scanning

After training in the MRI scanner, subjects were tested in a single session consisting of 20 trials each for the three trained RefTC exemplars randomly presented with 60 trials of RTC and 120 trials of NTC (which were generated anew). The task was the same as during training: on each trial after listening to a 1.5 s long stimulus, subjects indicated by button presses if the stimulus was repeated. The inter stimulus interval was 3 s. While the subjects were being tested, high-resolution fMRI data were continuously acquired. These data were our main focus and were analysed using MVPA. After the acquisition of functional data, a high-resolution structural scan was acquired in the same session. After the MRI scanning, listeners were debriefed by means of a questionnaire. In particular, a question was asked as to whether they thought that any of the sounds recurred during the experiment. One listener said yes, one said some and the remaining five said no.

### (e) Magnetic resonance imaging data acquisition

All imaging data were acquired on a Siemens 3 T Allegra head only scanner operated with a standard transmit-receive head coil. Functional data (T2^*^ weighted) were continuously acquired using single-shot high-resolution echo-planar imaging sequence (in-plane resolution = 1.5 × 1.5 mm^2^, field of view = 192 × 192 mm, matrix = 128 × 128, echo time (TE) = 30 ms, asymmetric echo shifted forward by 26 phase-encoding lines, echo spacing = 560 µs). Forty-two interleaved slices, repetition time (TR) 4.2 s, covering auditory cortex (HG, PT), STS and structures in the MTL (HC, PHC and EPC) were acquired. For correction of distortions in the magnetic field, field maps were acquired with a standard manufacturer's double-echo gradient echo field map sequence (TE = 10.0 and 12.46 ms, TR = 1020 ms, matrix size = 64 × 64) with 64 slices covering the whole head (voxel size = 3 mm isotropic). A high-resolution T1-weighted structural MRI scan (voxel size = 1 mm isotropic) was also acquired for each participant after the functional data collection.

### (f) Pre-processing of functional magnetic resonance imaging data

Pre-processing of the fMRI data was carried out using SPM8 (http://www.fil.ion.ucl.ac.uk/spm/software/spm8/). After discarding the first six volumes to allow for magnetic saturation effects, the remaining images were realigned to correct for movement of subjects during scanning. The images were then minimally smoothed with a 3 mm full width at half maximum Gaussian kernel. Each trial was modelled as a separate regressor where the listening time of each trial was modelled as an event and convolved with the canonical haemodynamic response function. Participant-specific movement parameters were included as regressors of no interest.

### (g) Region of interest segmentation

**Figure 2.**
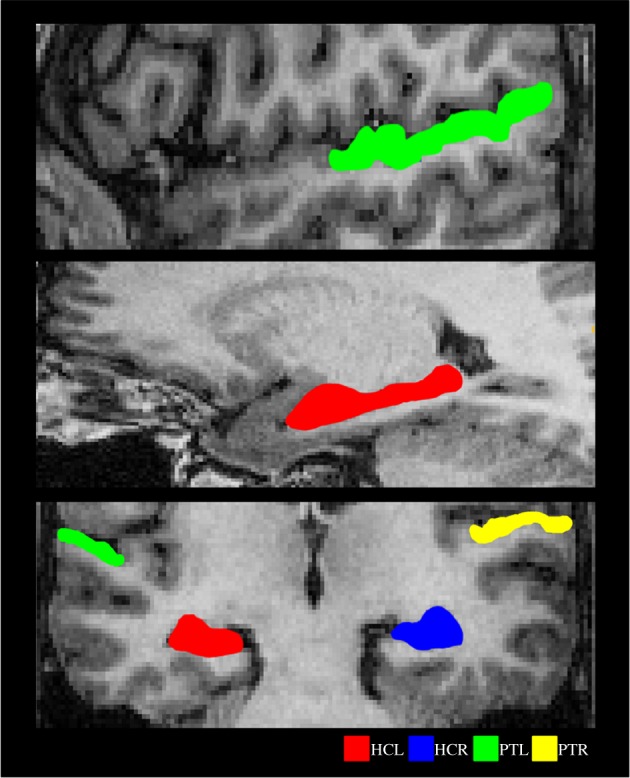
Examples of segmented ROIs. Shown on sagittal (upper and middle panel) and coronal (lower panel) sections from a subject chosen at random. HCL, left hippocampus; HCR, right hippocampus; PTL, left planum temporale; PTR, right planum temporale.

The structural scan of each participant was manually segmented using ITK-SNAP 2.2 [19] to delineate six regions of interest: HG, PT, STS, HC, PHC and EPC. Examples of segmentations for PT and HC are shown in figure 2. Segmentation of a structure in each hemisphere was done based on the landmarks and boundaries of that structure in an individual hemisphere.

Volumes of HG and PT were defined using the definitions of borders developed in [20,21]. The anterior border of the HG was defined by the complete transverse sulcus (TS), whereas the posterior border was defined using the complete Heschl's sulcus (HS). If there was a repetition of HG, only the anterior gyrus was considered and the posterior was included as part of PT. The postero-medial boundary of the HG was drawn on the axial section by drawing a line from the medial end of TS to the medial end of HS. The lateral boundary of the HG was defined by the termination of HG at the lateral surface of the superior temporal gyrus. The inferior boundary was demarcated in coronal section by noting the stem of HG in that section.

For segmenting PT, the posterior border of HG (Heschl's sulcus) was taken as the anterior border of PT. The markers for the posterior boundary of PT are not well established, because in most cases the posterior end of the sylvian fissure bifurcates into ascending and descending rami, the pattern of which varies across subjects [21]. There is no consensus on whether the posterior end of PT is limited to the posterior end of the horizontal portion of sylvian fissure [22,23] or extends all the way up to the end-point of the ascending ramus (which could cover a part of the parietal lobe [21]). In this study, we chose the latter option.

For STS, both upper and lower banks were marked on the coronal section. In subjects where the STS was interrupted by short gyri (‘plis de passage’, [24]) and ascended to the parietal lobe, all parts (anterior, middle, posterior, ascending anterior and ascending posterior, [24]) were included in the STS volume.

Hippocampal anatomy was identified using the Duvernoy HC atlas [25]. The EPC and PHC were segmented according to the protocol described in [26]. Mean volumes (in cubic millimetre, summed across both hemispheres) and standard deviations (SD) for the region of interests (ROIs) were as follows: HC 4188.8 (472.97), EPC 5026.04 (488.71), PHC 1799.29 (256.69), HG 2317.93 (521.93), PT 3967.07 (719.66) and STS 14396.07 (2476.29).

We initially used standard univariate analyses to interrogate the data, but did not find any significant difference in the mean activity evoked by the three RefTC stimuli in any part of the brain. We therefore focused on using MVPA which we believed would have increased sensitivity in our experimental context.

### (h) Multi-voxel pattern analysis

A linear support vector machine (SVM) classifier was created for each ROI. Each classifier was trained on a portion of the fMRI data relating to the three different exemplars of RefTCs and then tested on an independent set of instances of these exemplars.

We used a standard MVPA procedure that has been described in detail elsewhere [27,28] (for an in depth review, see [29]). The overall classification procedure involved splitting the fMRI data into two segments: a ‘training’ set used to train a classifier with fixed regularization hyperparameter *C* = 1, and a ‘test’ set used to independently test the classification performance using a standard 10-fold cross-validation testing procedure. This therefore generated 10 sets of SVM training and test sets that produced overall classification accuracy from the proportion of correct classification ‘guesses’ across all 10-folds of the cross-validation. The classification was performed using the LIBSVM implementation [30]. Prior to multivariate classification, feature selection [31] was performed on the data from the training set. This was conducted using a standard multivariate searchlight strategy within the given ROI.

### (i) Feature selection for multi-voxel pattern analysis

The purpose of feature selection is to reduce the set of features (in this case, voxels) in a dataset to those most likely to carry relevant information. This is effectively the same as removing voxels most likely to carry noise and is a way of increasing the signal-to-noise ratio. This was conducted using a standard multivariate searchlight strategy within the given ROI. For a given voxel, we first defined a small sphere with a radius of three voxels centred on a given voxel [27,32,33]. Note that the spheres were restricted so that only voxels falling within the given ROI were included. Therefore, the shape of the sphere and the number of voxels within it varied depending on the proximity to the ROI's borders. This procedure then allowed the selection of the searchlight voxel set that contained the greatest degree of decoding information within the training dataset. Using this voxel subset, the SVM classifier was trained to discriminate between the three RefTCs using the ‘training’ dataset, and tested on the independent ‘test’ dataset.

Standard SVMs are binary classifiers that operate on two-class discrimination problems, whereas our data involved a three-class problem (i.e. three exemplars). The SVM can, however, be arbitrarily extended to work in cases in which there are more than two classes. Typically, this is done by reducing the single multiclass problem into multiple binary classification problems that can be solved separately and then recombined to provide the final class prediction [34]. We used the well-established error correcting output codes approach [35] and computing of the Hamming distance [27,32,36]. The classifier accuracy values for each brain region were compared to chance, which in this case was 33% as we were classifying between three exemplars. Given that we were interested in whether results were significantly above chance, one tailed *t*-tests were used. Repeated measures ANOVAs were used to compare accuracy values between regions, and subsequently interrogated using two-tailed paired *t*-tests. A threshold of *p* < 0.05 was employed throughout. Since the training phase and the testing phase used different paradigms (during training separate RefTCs were presented in different blocks and in testing the three learned exemplars of RefTCs were presented in the same block), the fMRI data from the training phase were not analysed, and we focused on our main question of where the learned RefTCs were represented.

## 3. Results

### (a) Behavioural performance

**Figure 3.**
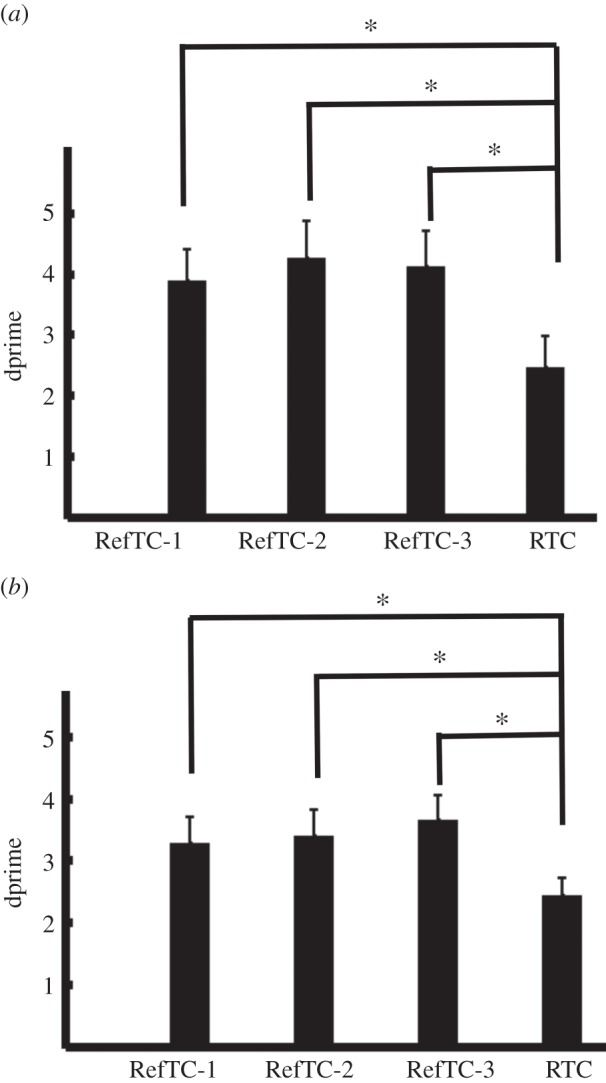
Behavioural performance during (*a*) training and (*b*) testing. dprimes (mean ± s.e. of mean) are plotted for the three exemplars that were repeated during the course of experiment (RefTC) and the exemplars that were the same structure as RefTC but were generated anew in each trial (RTC). RefTC-1, repeated reference exemplar 1; RefTC-2, repeated reference exemplar 2; RefTC-3, repeated reference exemplar 3; RTC, repeated stimulus. (*p < 0.05.)

Behavioural performance during training and testing is shown in figure 3. There was a significant effect of stimulus on performance during training (*F*_3,18_ = 7.08, *p* = 0.002). Post hoc analysis showed that dprimes for the three RefTC were greater than RTC (RefTC-1 > RTC: *t*_6_ = 2.93, *p* = 0.02; RefTC-2 > RTC: *t*_6_ = 5.12, *p* = 0.002; RefTC-3 > RTC: *t*_6_ = 2.97, *p* = 0.02). Performance on the three RefTC did not differ significantly. Analysis of behavioural performance during testing showed a significant effect of stimulus on dprimes (*F*_3,18_ = 8.98, *p* = 0.001). Post hoc comparison revealed better performance on all the RefTC stimuli compared to RTC (RefTC-1 > RTC, *t*_6_ = 3.96, *p* = 0.007; RefTC-2 > RTC, *t*_6_ = 4.30, *p* = 0.005; RefTC-3 > RTC, *t*_6_ = 5.43, *p* = 0.001). Thus, consistent with previous findings [5,6,37], the behavioural data show learning of spectro-temporal patterns that are repeatedly presented during the course of the experiment. The novel question we then addressed was where in the brain these RefTCs were represented.

### (b) Multi-voxel pattern analysis

Using MVPA we examined whether it was possible to discriminate which of the three RefTC stimuli was being heard solely from the pattern of activity across voxels in our ROIs. For each ROI, a linear SVM classifier was first trained on a portion of the fMRI data relating to the three RefTC stimuli and then tested on an independent set of trials of these stimuli. If information was present in the patterns of fMRI activity that would allow for successful discrimination between the three stimuli, then the classifier would produce a result that was significantly above chance (33%). There was no statistically significant difference between the classification accuracies of left and right hemispheres (HC: *t*_6_ = 0.465, *p* = 0.658; EPC: *t*_6_ = −1.666, *p* = 0.147; PHC: *t*_6_ = −0.511, *p* = 0.628; HG: *t*_6_ = −1.384, *p* = 0.216; PT: *t*_6_ = −1.532, *p* = 0.177; STS: *t*_6_ = 0.789, *p* = 0.460) and therefore the reported data are collapsed across hemispheres.

**Figure 4.**
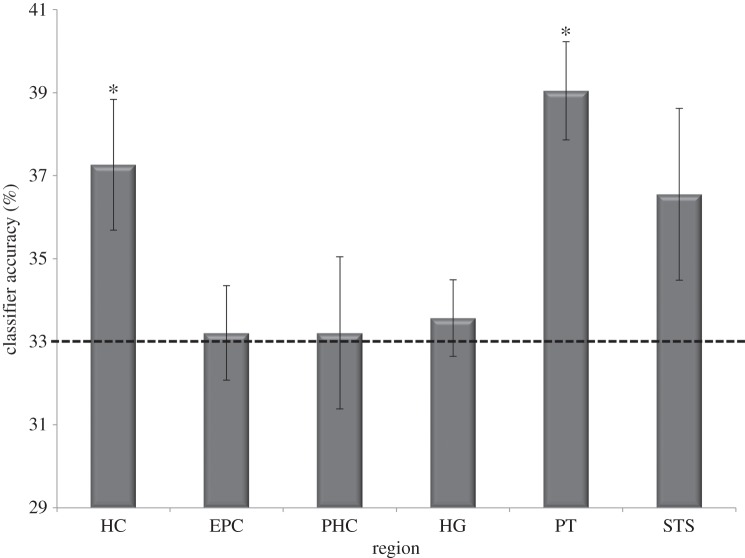
Results of the MVPA analysis. The mean (±s.e. of mean) classifier performance (collapsed across both hemispheres) is shown. Chance was 33% (marked by the dotted line). HC, hippocampus; EPC, entorhinal/perirhinal cortex; PHC, parahippocampal cortex; HG, Heschl gyrus; PT, planum temporale; STS, superior temporal sulcus. (*p < 0.05.)

Classification accuracy for the six ROIs is shown in figure 4. Only two regions, HC and PT, showed performance above chance: (HC: *t*_6_ = 2.711, *p* = 0.018; EPC: *t*_6_ = 0.188, *p* = 0.43; PHC: *t*_6_ = 0.117, *p* = 0.46; HG: *t*_6_ = 0.620, *p* = 0.28; PT: *t*_6_ = 5.106, *p* = 0.001; STS: *t*_6_ = 1.715, *p* = 0.07). A repeated measures ANOVA showed a significant effect for region (*F*_5,30_ = 3.271, *p* = 0.018), which was driven by more information being present in the HC when compared with HG (HC > HG: *t*_6_ = 2.765, *p* = 0.033) as well as more information present in PT when compared with HG, EPC and PHC (PT > HG: *t*_6_ = 4.182, *p* = 0.006; PT > EPC: *t*_6_ = 3.059, *p* = 0.022; PT > PHC: *t*_6_ = 3.291, *p* = 0.017). Classification accuracy in the STS was almost significant (see above) and did not differ significantly from either PT (*t*_6_ = 1.91, *p* = 0.11) or HC (*t*_6_ = 0.25, *p* = 0.81).

To ensure that the classifiers were unbiased and that classification was based on unique representations for each of the three RefTCs, we ran a control analysis in which we randomly shuffled (from trial to trial) the labels of the three RefTCs. The classifiers were trained and tested as above. As expected, the performance of the classifier dropped to chance level for all the brain regions (HC: *t*_6_ = −0.341, *p* = 0.628; EPC: *t*_6_ = 0.735, *p* = 0.245; PHG: *t*_6_ = 1.047, *p* = 0.168; HG: *t*_6_ = 0.392, *p* = 0.354; PT: *t*_6_ = 0.109, *p* = 0.459; STS: *t*_6_ = −0.147, *p* = 0.557).

To further confirm that classification of RefTCs was based on stable representations (owing to the repeated presentation of the same exemplars), we ran a second control analysis. In this analysis, we divided the 60 RTC trials randomly into three classes and performed the same classification analysis. Since a different exemplar is presented in every trial of RTC, representation of RTC changes from trial to trial. It is therefore expected that the classifier should not be able to classify the RTC stimuli significantly better than chance. As predicted, the classifier performance was at chance for all ROIs, (HC: *t*_6_ = −0.058, *p* = 0.955; EPC: *t*_6_ = −0.421, *p* = 0.689; PHG: *t*_6_ = 0.953, *p* = 0.377; HG: *t*_6_ = 1.365, *p* = 0.221; PT: *t*_6_ = −0.174, *p* = 0.868; STS: *t*_6_ = −0.316, *p* = 0.763).

## 4. Discussion

As in Agus *et al.* [5], the behavioural data in our study revealed better performance on repeated exemplars compared to performance on non-repeated exemplars confirming learning of acoustic patterns. Importantly, the repeatedly presented stimuli (RefTCs) and the non-repeated stimuli (RTC) were well balanced with respect to acoustic parameters. The key difference between the two conditions was in terms of exposure over the course of the experiment. Subjective reports from the participants after the experiment showed that most were not aware of any stimulus re-occurring during the experiment, suggesting that in some cases the learning occurred implicitly.

We found that patterns of activity across voxels in PT, but not in HG, could distinguish between the three learned acoustic patterns. We speculate that this might reflect a type of representation within PT that is not a simple representation of acoustic pattern but a more refined representation that requires interaction with HC (see below). The availability of pattern specific information in the PT is consistent with a role as a computational hub [12], where spectro-temporal patterns are matched with learned patterns that are stored beyond the auditory cortex. Our results showing the involvement of auditory cortex in the storage of long-term representations of stimuli without semantic association is consistent with a recent magnetoencephalography study [37] that showed different phase patterns for different noise exemplars that had been learnt, suggesting a specificity of the representation for a given pattern. However, compared to the results in [37], our results are more specific with respect to the areas of auditory cortex involved in storing pre-semantic-specific templates.

Our results show that HC is involved in representations of RefTC stimuli that are unique to each exemplar of RefTC. Although HC is known to be involved in processing of complex and meaningful sounds [15,38], to the best of our knowledge, our study is the first to demonstrate the encoding of noise-like acoustic patterns in the HC. There are some studies in the visual domain [39] which showed sensitivity of the HC to changes in low-level perceptual features (e.g. change in font size of displayed letters). The results of our study further extend these results by showing that the HC is not only sensitive to low-level features of stimuli that are explicitly recalled, but also constructs representations that are specific to acoustic features which are learned implicitly.

Our results also showed that the classifier performance was close to significant (*p* = 0.07) for the STS region. This lack of significance may be owing to the low power of our study, so a role for STS in the learning of novel acoustic patterns cannot be ruled out. The STS is a broad region which has been implicated in a wide range of unimodal and multimodal functions (for review, see [40]). From the auditory perception point of view, converging evidence from neuroimaging studies show that STS is involved in categorical perception of speech [13] and non-speech [14,41] auditory stimuli. Categorical perception involves mapping a continuum of variation for low-level acoustic features of the stimuli into a discrete number of abstract categories. Furthermore, the connectivity analysis [42] between the HG, PT and STS shows that representations of acoustic features in HG and PT are relayed to the STS for object like representations. In the context of current study, it is therefore likely that although each of the three RefTC's has a unique representation in the PT, these representations may have been further abstracted and instantiated in the STS.

We considered whether our results might be explained by sensory or perceptual representations in PT and/or HC. The existence of sensory representations of fine spectro-temporal features that occurs in non-primary auditory cortex and HC but not in primary auditory cortex is unlikely. Mapping of the different perceptual timbre of different exemplars could occur in non-primary auditory cortex, but there is no precedent for any effect of manipulating spectro-temporal structure and timbre on hippocampal activity [43–46]. The most parsimonious explanation for our data is a unique memory trace in PT and HC that is formed for each of the three RefTCs, although more work will be needed to probe this point further. It will also be important to examine another issue in the future. Our learning phase was optimized to facilitate acquisition of the three RefTCs; consequently, the fMRI data acquired during this phase were not intended (nor were they suitable) for analysis using MVPA, because our focus was solely on where the learnt RefTCs were represented. Further studies could investigate the learning over time of RefTCs to see if PT and HC (and/or other brain regions) are implicated.

Structural connectivity between human auditory cortex and the MTLs is not completely understood. However, the connectivity pattern between auditory cortex and the MTL of monkeys [47] (for review, see [17]) shows that belt and parabelt, but not the core, of the auditory cortex have direct projections to the entorhinal cortex. The human PT we examined in our experiment is a non-core area containing homologues of primarily auditory belt (and possibly auditory parabelt) cortex [48]. Based on the non-human primate work, therefore, it appears likely that PT and HC are reciprocally connected. The connectivity between PT and HC offers good grounds for proposing that PT and HC might form a connected system that allows specific representations of learnt spectro-temporal patterns. The purpose of such a system may be to transform the rich pattern of activity for complex sounds expressed in HG into sparser representations, more amenable to long-term memory storage. This raises questions about the dynamics of this system, as behavioural data show that learning occurs rapidly. Further work is required to determine how many repetitions of each exemplar are required during the training phase before a stable representation is built in the PT and HC. A further question concerns the direction of causal influences of the PT and HC on each other that occur during the construction of stable representations. As a first step, this study establishes the existence of stabilized representations of sound structure in PT and HC concurrent with auditory learning.

## Notes

The study was approved by the local research ethics committee.

## Data accessibility

The procedure we have agreed with the UCL Ethics Committee is that any request for the data needs to be initiated from an appropriate source (usually a fellow scientist) after which we will make the anonymized data available to him or her.
